# ChIP-seq Analysis of the Global Regulator Vfr Reveals Novel Insights Into the Biocontrol Agent *Pseudomonas protegens* FD6

**DOI:** 10.3389/fmicb.2021.667637

**Published:** 2021-05-14

**Authors:** Qingxia Zhang, Chenglin Xing, Xiangwei Kong, Cheng Wang, Xijun Chen

**Affiliations:** ^1^College of Horticulture and Plant Protection, Yangzhou University, Yangzhou, China; ^2^Joint International Research Laboratory of Agriculture and Agri-Product Safety, The Ministry of Education of China, Yangzhou University, Yangzhou, China

**Keywords:** *Pseudomonas protegens*, 2, 4-DAPG, biological control, Vfr, antibiotic

## Abstract

Many *Pseudomonas protegens* strains produce the antibiotics pyoluteorin (PLT) and 2,4-diacetylphloroglucinol (2,4-DAPG), both of which have antimicrobial properties. The biosynthesis of these metabolites is typically controlled by multiple regulatory factors. Virulence factor regulator (Vfr) is a multifunctional DNA-binding regulator that modulates 2,4-DAPG biosynthesis in *P. protegens* FD6. However, the mechanism by which Vfr regulates this process remains unclear. In the present study, chromatin immunoprecipitation of FLAG-tagged Vfr and nucleotide sequencing analysis were used to identify 847 putative Vfr binding sites in *P. protegens* FD6. The consensus *P. protegens* Vfr binding site predicted from nucleotide sequence alignment is TCACA. The qPCR data showed that Vfr positively regulates the expression of *phlF* and *phlG*, and the expression of these genes was characterized in detail. The purified recombinant Vfr bound to an approximately 240-bp fragment within the *phlF* and *phlG* upstream regions that harbor putative Vfr consensus sequences. Using electrophoretic mobility shift assays, we localized Vfr binding to a 25-bp fragment that contains part of the Vfr binding region. Vfr binding was eliminated by mutating the TACG and CACA sequences in *phlF* and *phlG*, respectively. Taken together, our results show that Vfr directly regulates the expression of the 2,4-DAPG operon by binding to the upstream regions of both the *phlF* and *phlG* genes. However, unlike other Vfr-targeted genes, Vfr binding to *P. protegens* FD6 does not require an intact binding consensus motif. Furthermore, we demonstrated that *vfr* expression is autoregulated in this bacterium. These results provide novel insights into the regulatory role of Vfr in the biocontrol agent *P. protegens*.

## Introduction

Pseudomonads are gram-negative bacteria that are widely distributed in the rhizosphere; some strains of pseudomonads are members of plant microbiomes that contribute to plant growth and disease suppression. Several studies have suggested that antifungal metabolites produced by *Pseudomonas* spp. including phloroglucinols, phenazines, pyoluteorin, pyrrolnitrin, lipopeptides and hydrogen cyanide, exert biological control of plant diseases (Haas and Keel, 2003; Keswani et al., 2020). 2,4-Diacetylphloroglucinol (2,4-DAPG) is a broad-spectrum antibiotic that is toxic to various plant pathogens, including bacteria, fungi, oomycetes, and nematodes (Haas and Defago, 2005). The 2,4-DAPG biosynthesis gene cluster includes eight genes that are collectively named *phlACBDEFGH* (Bangera and Thomashow, 1999). The four structural genes constitute a single operon (*phlACBD*) and are directly involved in 2,4-DAPG biosynthesis. PhlF is a TetR family regulator that is involved in the regulation of 2,4-DAPG production. PhlF, which functions upstream of PhlA, impedes the initiation of *phlACBD* operon transcription by binding to the promoter region upstream of *phlA* (Abbas et al., 2002). PhlG specifically degrades 2,4-DAPG to less toxic monoacetylphloroglucinol (MAPG) (Bottiglieri and Keel, 2006), and 2,4-DAPG functions as an autoinducer that activates *phlACBD* operon transcription (Schnider-Keel et al., 2000). In addition, 2,4-DAPG production is typically regulated by many complex regulatory systems (Yan et al., 2017).

Cyclic AMP (cAMP) receptor proteins (CRPs) are global transcriptional regulators that are broadly distributed in various bacterial species. CRPs are among the best-studied transcriptional regulators in *Escherichia coli* and can modulate the expression of more than 180 genes in a cAMP-dependent manner (Kolb et al., 1993; Soberón-Chávez et al., 2017). In *E. coli*, the CRP/cAMP dimer is able to bind a conserved 22-bp DNA sequence (5’-AAAT**G**T**G**AN6T**C**A**C**ATTT-3’) (Berg and von Hippel, 1988) that harbors two conserved DNA binding motifs (these are underlined in the foregoing sequence). A binding sequence for Vfr (5’-ANWWTGNGAWNYAGWTCACAT-3’) has also been identified in *P. aeruginosa*; this sequence includes two conserved half-sites (underlined) that are similar to those of CRP. However, in Vfr-dependent promoters, the TCACA motif is more conserved (Kanack et al., 2006).

The transcription factor Vfr regulates the expression of genes that are important for *P. aeruginosa* virulence, including those associated with type IV pili, extracellular polysaccharides, the flagellum, exolysin expression and cytotoxicity (Berry et al., 2018). The role of Crp family regulators in pathogenicity has been demonstrated in phytopathogenic bacteria. The cAMP receptor-like protein (Clp) of *Xanthomonas campestris* pv. *campestris* regulates the expression of 299 genes and is required for diffusible signal factor regulation of virulence factor production (He et al., 2007). Furthermore, in *P. syringae* pv. *tabaci* 6605, Vfr was shown to control virulence-associated phenotypes in a quorum sensing-independent manner (Taguchi and Ichinose, 2013). In addition to their roles in pathogenic bacteria, the roles of CRP homologs in biocontrol agents have been fully elucidated. For example, Clp not only controls the production of HSAF and extracellular chitinase but also modulates twitching motility in *Lysobacter enzymogenes* OH11 (Wang et al., 2014; Xu et al., 2016). In *P. protegens* FD6, Vfr negatively regulates the synthesis of the antibiotics 2,4-DAPG and pyoluteorin (PLT) (Zhang et al., 2016), although the mechanism by which Vfr regulates gene expression has not been fully elucidated.

*Pseudomonas protegens* FD6 is a biocontrol strain obtained from the canola rhizosphere in Fujian province, China that produces a number of secondary metabolites, including pyrrolnitrin, pyoluteorin, 2,4-DAPG, extracellular protease, siderophore and hydrogen cyanide (Chang et al., 2011). The antibiotics 2,4-DAPG and pyoluteorin have been shown to be the primary contributors to the ability of *P. protegens* FD6 to inhibit the growth of phytopathogenic fungi (Zhang et al., 2020). The hybrid sensor kinase RetS and Vfr negatively control antibiotic biosynthesis in *P. protegens* FD6; the former functions through the Gac/Rsm pathway (Zhang et al., 2015, 2016). However, the molecular mechanism by which Vfr regulates antibiotic synthesis in *P. protegens* has remained unknown.

Two major high-throughput approaches are used to identify the binding sites of a specific transcription factor. One approach involves the use of elegant computational methods to perform homology searches. The other approach, which is based on the use of chromatin immunoprecipitation (ChIP) and ChIP-sequencing (ChIP-seq), has been shown to be a powerful tool for identifying bacterial regulons in various bacterial species (Myers et al., 2013; Perkins et al., 2013; Kleinman et al., 2017; Tsai et al., 2018). In the present study, no Vfr target genes were identified when nine conserved residues of the Vfr binding motif were used to search the complete *P. protegens* FD6 genome. We then constructed mutants expressing FLAG-tagged Vfr in a *vfr* deletion mutant and performed ChIP-seq *in vivo* to assess the interaction of Vfr with the chromosome. Based on these results, two novel Vfr-regulated genes were identified and shown to be involved in controlling 2,4-DAPG synthesis. Our results revealed that Vfr acts as an activator or as a repressor to modulate the expression of approximately 847 genes that are involved in a variety of physiological processes. These results supply a comprehensive map of Vfr binding sites across the *P. protegens* genome and provide novel insights into Vfr-related global gene expression in this biocontrol agent.

## Materials and Methods

### Bacterial Strains, Plasmids, and Growth Conditions

All of the bacterial strains and plasmids used in this study are listed in Table 1. *P. protegens* strains were cultured at 30°C in Luria-Bertani (LB) medium. *Escherichia coli* strains were routinely grown at 37°C in LB medium. *P. protegens* cells were electroporated using a Bio-Rad Gene Pulser II (Bio-Rad, CA, United States) at 1.8 kV and 300 Ω. Antibiotics were used at the following concentrations: kanamycin 50 μg mL^–1^; ampicillin 50 μg mL^–1^; streptomycin (Str) 16 μg mL^–1^; tetracycline 15 μg mL^–1^; gentamycin 10 μg mL^–1^ (for *E. coli*), or 50 μg mL^–1^ (for *P. protegens*); and chloramphenicol 34 μg mL^–1^.

**TABLE 1:** Bacterial strains and plasmids used in this study.

**Strains and plasmids**	**Characteristics**	**Reference or source**
**Strains**		
*Escherichia coli*		
DH5α	F^–^ *recA1 endA1 hsdR17 deoR thi-1 supE44 gyrA96 Re lA1(lacZYA-argF)U169ë-(Ö80dlacz △M15)*	Hanahan, 1983
XL1-Blue MRF’ Kan	Δ (*mcrA*) *183*,Δ (*mcrCB-hsdSMR-mrr*) *173, endA1,supE44, thi-1,recA1 gyrA96, relA1, lac*, [F’*proAB lacIqZ*ΔM15 Tn5 (Kan^*r*^)]	Stratagene
BL21(DE3)	F^–^ *omp*T *hsdSB* (rB^–^ mB^–)^ *gal dcm met* (DE3)	Novagen
*P. protegens*		
FD6	Wild type; Ap^*r*^	Chang et al., 2011
Δvfr	*vfr* gene in-frame deletion in strain FD6; Ap^*r*^	Zhang et al., 2016
Δvfr/pBBR-vfr-3FLAG	Mutant Δvfr harboring plasmid pBBR-vfr-3FLAG; Km^*r*^	This study
Δvfr/pBBR-3FLAG	Mutant Δvfr harboring plasmid pBBR-3FLAG; Km^*r*^	This study
Δvfr/pBBR	Mutant Δvfr harboring plasmid pBBR; Km^*r*^	This study
**Plasmids**		
pET22b(+)	f1 origin, expression vector; Ap^*r*^	Novagen
pTRG	Plasmid used for protein expression in the bacterial one-hybrid assay; Tet^*r*^	Stratagene
pBXcmT	Plasmid used for DNA cloning in the bacterial one-hybrid assay; Cm^*r*^	Guo et al., 2009
pBBR1MCS-2	Broad-host-range cloning vector; Km^*r*^	Kovach et al., 1995
pET22b-vfr	pET22b with 645 bp fragment including the *vfr*;Ap^*r*^	This study
pTRG-vfr	pTRG with the coding region of the *vfr*; Tet^*r*^	This study
pBXcmT-vfr	pBXcmT with putative Vfr binding sites of *vfr*; Cm^*r*^	This study
pBXcmT-phlF	pBXcmT with putative Vfr binding sites of *phlF*; Cm^*r*^	This study
pBXcmT-phlG	pBXcmT with putative Vfr binding sites of *phlG*; Cm^*r*^	This study
pUC57-vfr-3FLAG	pUC57 with 3 × FLAG tagged Vfr; Ap^*r*^	This study
pBBR-vfr-3FLAG	pBBR1MCS-2 carrying intact vfr-3 × FLAG sequence; Km^*r*^	This study
pBBR-3FLAG	pBBR1MCS-2 carrying intact 3 × FLAG sequence; Km^*r*^	This study

### ChIP

For ChIP-seq studies, Vfr was tagged at the carboxy terminus with a 3 × FLAG epitope tag. Briefly, Vfr fused with the 3 × FLAG epitope was synthesized and inserted into pUC57 to generate the plasmid pUC57-vfr-3FLAG. The plasmid was sequenced by General Biosystems, Inc. to ensure its correctness. pUC57-vfr-3FLAG was digested with *Kpn*I and *Hin*dIII (Takara, Japan) and ligated into the shuttle plasmid pBBR1MCS-2 to generate the plasmid pBBR-vfr-3FLAG. After confirmation by restriction digestion and sequencing, the recombinant plasmid pBBR-vfr-3FLAG was introduced into a *P. protegens* Δvfr mutant by electroporation. The resulting strain (Δvfr/pBBR-vfr-3FLAG) harbored a multicopy plasmid with tagged Vfr and was used in subsequent ChIP-seq studies. The functionality of the tagged Vfr in the Δvfr mutant was verified by Western blot analysis using an M2 monoclonal anti-FLAG antibody (Sigma, MO, United States). To construct a pBBR-3FLAG, the 3 × FLAG fragment was obtained by PCR amplification using the primers Flag-infusion-F/Flag-infusion-R and the plasmid pBBR-vfr-3FLAG as template. Linearized pBBR1MCS-2 was obtained by PCR amplification using the primers pBBR-R-F/pBBR-R-R, and the 3 × FLAG fragment was inserted into the linearized pBBR1MCS-2 vector through one-step cloning, generating the plasmid pBBR-3FLAG for chromatin immunoprecipitation PCR analysis (ChIP-PCR). The plasmid pBBR-3FLAG was also transformed into the Δvfr strain by electroporation, and this transformed strain served as the “mock” control. For ChIP-seq studies, the 3 × FLAG-tagged Vfr-harboring strain (Δvfr/pBBR-vfr-3FLAG) was grown overnight at 28°C in LB medium and then subcultured in 50 mL of LB broth to mid-log phase (OD_600_ of approximately 0.6). Proteins bound to DNA were crosslinked with formaldehyde (1% final concentration) at room temperature for 20 min, and the reaction was quenched for 10 min with 0.125 M glycine. After one wash with 20 mL of phosphate-buffered saline (PBS), the cells were resuspended in 0.5 mL of FA lysis buffer (50 mM HEPES-KOH (pH 8.0), 140 mM NaCl, 1 mM EDTA, 1% Triton X-100, 0.1% sodium deoxycholate, and 10 mg mL^–1^ lysozyme) supplemented with an EDTA-free protease inhibitor cocktail (Roche, BW, Germany). Their genomic DNA was sheared by sonication to fragments 0.1–0.5 kb in size. Bacterial debris was removed by centrifugation at 12,000 rpm for 20 min, and a fraction of the supernatant was stored as the input sample for IP assays. ChIP was performed as described in a previous study (Bonocora and Wade, 2015; Xu et al., 2016). For the ChIP assays, a mixture of 200 μL of fragmented chromatin and 800 μL of IP buffer (50 mM HEPES-KOH (pH 7.5), 150 mM NaCl, 1 mM EDTA, 1% Triton X-100, 0.1% sodium deoxycholate, 0.1% SDS, and 1 mM PMSF) was incubated with 20 μL of ANTI-Flag^®^ M2 Affinity Gel (Sigma, MO, United States) at 4°C overnight on a rotator as previously described. The beads were then collected by centrifugation and washed sequentially with IP buffer and wash buffer (10 mM Tris-HCl (pH 8.0), 250 mM LiCl, 1 mM EDTA, 0.5% Triton X-100, and 0.5% sodium deoxycholate). The immunoprecipitated chromatin was removed from the beads by adding 100 μL of elution buffer (50 mM Tris-HCl (pH 7.5), 10 mM EDTA, and 1% SDS). The isolated complexes were then incubated at 65°C overnight to reverse the cross-linking. The samples were then treated with RNaseA, and the immunoprecipitated proteins were digested with proteinase K. The DNA was then cleaned using a Qiagen Mini Reaction Cleanup kit, and the DNA concentrations were determined using a Quibit^®^ 3.0 Fluorometer (Thermo Fisher Scientific, MA, United States).

Immunoprecipitated DNA was used to construct sequencing libraries according to the protocol provided with the NEXTFLEX^®^ ChIP-Seq Library Prep Kit for Illumina^®^ Sequencing (NOVA-514120, Bioo Scientific, Beijing, China). High-throughput sequencing was performed using the HiSeq X Ten Sequencing System (Illumina, CA, United States) at Wuhan IGENEBOOK Biotechnology Co., Ltd. The clean reads were aligned to the genomic DNA sequence of *P. protegens* FD6 (accession number CP031396) using the Burrows-Wheeler aligner method (Li and Durbin, 2009). Peak calling was performed with MACS2 (version 2.1.1.20160309) (Salmon-Divon et al., 2010), and HOMER (version 3) was used to predict motif occurrence within peaks using the default settings for a maximum motif length of 12 base pairs (Hull et al., 2013). All of the raw sequencing data from the ChIP-seq experiments have been deposited in the NCBI database under the accession number PRJNA649382.

### RNA Extraction and Quantitative Reverse Transcription PCR (RT-qPCR)

Total bacterial RNA was extracted from *P. protegens* using an SV total RNA extraction kit (Promega, WI, United States). RNA samples were treated with DNase I (Takara, Japan) according to the manufacturer’s instructions to remove any residual DNA. RNA samples were reverse-transcribed and used as templates for RT-qPCR with iTaq Universal SYBR Green Supermix (Bio-Rad, CA, United States). The *rrsB* gene was amplified as an internal control. A real-time PCR machine (CFX96 Touch^TM^, Bio-Rad, CA, United States) was used for RT-qPCR in 96-well plates with the following program: 3 min at 95°C followed by 40 cycles of 95°C for 10 s and 60°C for 30 s. Each RT-qPCR experiment was repeated three times, with three technical repeats per sample. The primers used for RT-qPCR analysis are described in Supplementary Table 1.

### Bacterial One-Hybrid Assays

The bacterial one-hybrid system was derived from the BacterioMatch II two-hybrid system (Stratagene) and has been shown to be efficient for assaying the potential interaction between a transcription factor and its target gene promoter (Guo et al., 2009; Xu et al., 2016). The bacterial one-hybrid reporter system contained three components: the *E. coli* XL1-Blue MRF’ kan host strain and the plasmids pTRG and pBXcmT, which were utilized to express the target proteins and clone their bait DNA, respectively. The reporter vector pBXcmT is derived from the pBT bait plasmid and contains the selectable genes *HIS3* and *aadA* (Guo et al., 2009). Therefore, the bacterial one-hybrid system, like the BacterioMatch II two-hybrid system, also uses a *HIS3*-*aadA* reporter cassette. In this system, detection of protein-DNA interactions is based on the transcriptional activation of both *HIS3* and *aadA*, both of which are located in the F’ factor of the reporter strains. This activation produces transformants that are able to grow on a medium lacking histidine and exhibit streptomycin resistance. As suggested in the instruction manual furnished with the BacterioMatch II Two-Hybrid System Vector Kit, *E. coli* XL1-Blue MRF’ kan containing the *lacI*^*q*^ gene was used as a host strain for propagation of the pTRG and pBXcmT plasmids to minimize the toxic effects of the bait and target proteins on the host. Vector construction was performed as previously described (Xu et al., 2016). The *vfr* gene was amplified using the primers pTRG-vfr-F/pTRG-vfr-R (Supplementary Table 1) and cloned into the *Eco*RI and *Xho*I sites of the pTRG vector. The Vfr binding sequences in the *P. protegens* FD6 genes were amplified using specific primers (Supplementary Table 1) and ligated into pBXcmT using the *Eco*RI and *Xba*I sites. Cotransformants showing positive growth were then selected, and *E. coli* XL1-Blue MRF9 Kan cotransformed with the pBXcmT and pTRG plasmids was tested as a negative control on selective screening medium containing 20 mM 3-AT (3-amino-1,2,4-triazole), Km^*r*^, Str^*r*^, Tet^*r*^, and Cm^*r*^. The plates were incubated at 30°C for 4–5 days.

### Protein Expression and Purification

C-terminal His_6_-tagged Vfr was expressed using the pET22b (+) vector in *E. coli* BL21 (DE3) cells. Briefly, cultures were grown to an OD_600_ of 0.6, and then induced with 0.5 mM isopropyl-β-D-thiogalactoside for 8 h at 20°C. The cells were then harvested by centrifugation, washed twice with 30 mL of suspension buffer (15 mM Tris-HCl (pH 6.5), 0.5 M NaCl, and 10% glycerol), and resuspended in 50 mL of suspension buffer. The cell suspensions were disrupted using an Ultrasonic cell crusher JY92-IIN (Scientz, Ningbo, China) with 3-s burst and 3-s rest periods until the suspension cleared and then centrifuged at 17,000 rpm for 40 min at 4°C. The 6 × His-tagged Vfr protein was purified using Ni-NTA beads (GenScript, Nanjing, China) according to the bead manufacturer’s instructions. Purified recombinant protein was concentrated using Amicon Ultra-15 concentrators (Millipore, MA, United States) and equilibrated in storage buffer (40 mM Tris-HCl (pH 7.2), 500 mM KCl, 0.2 mM EDTA, 0.2 mM DTT, and 50% glycerol) at –80°C until use. Protein concentrations were determined using a Genova Nano instrument (Bibby Jenway, Staffordshire, United Kingdom).

### Electrophoretic Mobility Shift Assays (EMSAs)

To detect Vfr-DNA binding, PCR products of the intergenic sequences upstream of the genes *vfr*, *phlF* and *phlG* were labeled with biotin (EMSA Probe Biotin Labeling Kit, Beyotime, Shanghai, China) and purified using the phenol-chloroform extraction method. The sequences of the DNA oligos are provided in Supplementary Table 1. Labeled DNA probe (1 μmol) and 0.5–4 μg of Vfr were used in 10-μL reactions according to the manufacturer’s protocol (Chemiluminescent EMSA Kit, Beyotime, Shanghai, China). The mixtures were incubated at 25°C for 20 min and then separated by 4% non-denaturing polyacrylamide gel electrophoresis at 100 V in 0.5 × Tris-borate-EDTA buffer (pH 8.0). In competition analyses, unlabeled probes were added prior to addition of the labeled probes. The gels were incubated with a streptavidin-HRP conjugate, and chemiluminescence was measured using Image Lab^TM^ (BIO-RAD ChemiDoc^TM^ XRS+, CA, United States).

### Western Blot Analysis

Bacteria were pelleted from 1-mL overnight cultures, and the cells were denatured by boiling in 5 × SDS loading buffer (200 mM Tris-HCl (pH 6.8), 10% SDS, 30% glycerol, 0.2% bromophenol blue, and 5% β-mercaptoethanol) for 5 min. Whole-cell extracts were then loaded and separated on 12.5% polyacrylamide gels and assessed by Western blot analysis as previously described (Chen et al., 2018). The blots were treated with monoclonal ANTI-FLAG^®^ M2, clone M2 (1:5,000) as the primary antibody and with AP-conjugated goat anti-mouse IgG as the secondary antibody. The blots were then developed using nitroblue tetrazolium and 5-bromo-4-chloro-3-indolyl-phosphate as the chromogenic substrate.

### Statistical Analysis

All of the qPCR data were examined for statistical significance using the independent samples *t*-test using SPASS 25.0.

## Results

### Identification of Vfr Binding Sites

To determine whether the Vfr-FLAG fusion protein was successfully expressed in the Δvfr mutant, we performed Western blotting using an anti-FLAG antibody. A 24.2- kDa band was clearly observed among the total proteins expressed by Δvfr/pBBR-vfr-3FLAG, whereas no band was observed in the negative control strain Δvfr/pBBR (Supplementary Figure 1). This result indicated that the FLAG-tagged Vfr is expressed *in vivo* and that it could be used in further ChIP-seq analysis.

**FIGURE 1:**
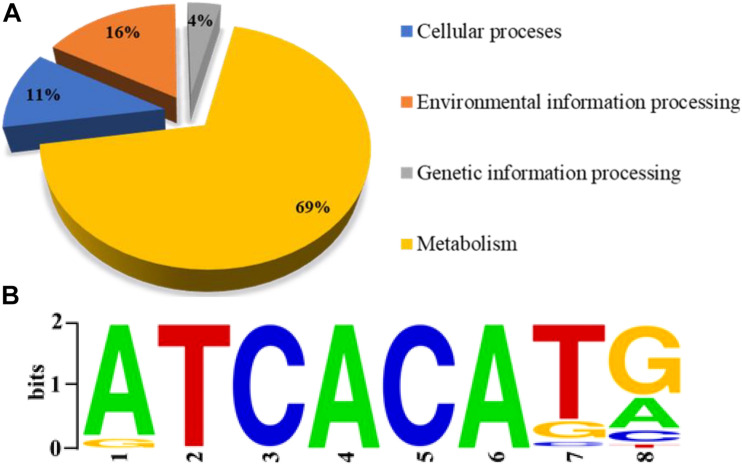
Genome-wide analysis of the Vfr regulon by chromatin immuno-precipitation sequencing (ChIP-seq). **(A)** Pie chart showing the shoeing of Vfr targets with functional categories defined in the *Pseudomonas* database (http://pseudomonas.com). **(B)** The most significant motif identified by ChIP-seq using the HOMER tool is shown. The height of each letter is proportional to the level of conservation at that site.

Sequence reads were obtained from two independent ChIP-seq tests using a FLAG-specific antibody and mapped to the *P. protegens* FD6 genome. The ChIP-seq peaks were annotated to 847 genes (*q* < 0.05) using MACS (Salmon-Divon et al., 2010). Approximately 35.51 and 64.49% of the binding sites were located in coding sequences and promoter regions, respectively (Supplementary Figure 2). Moreover, these loci were distributed across the genome, suggesting that Vfr is a global transcriptional regulator in *P. protegens.* To further elucidate the molecular functions of the identified target genes of Vfr, the genes were categorized based on KEGG pathway analysis. The products encoded by Vfr-bound genes have different functions, including functions associated with metabolism (69%), environmental information processing (16%), cellular processes (11%), and genetic information processing (4%) (Figure 1A). The ChIP-seq data allowed us to further define the consensus-binding site of Vfr. Using the HOMER tool (Hull et al., 2013) to analyze the identified peaks, we identified a 5-bp Vfr consensus sequence (5’-TCACA-3’; Figure 1B) matching the conserved motif elucidated for *E. coli* and *P. aeruginosa* (Berg and von Hippel, 1988; Kanack et al., 2006).

**FIGURE 2:**
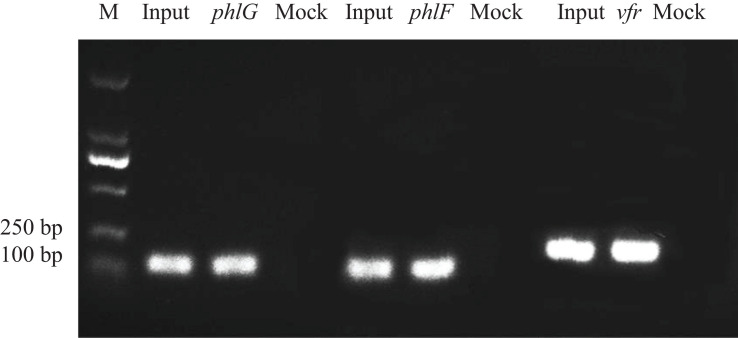
Chromatin immunoprecipitation polymerase chain reaction assays. Three specific bands representing putative Vfr binding sequences for *phlG*, *phlF* and *vfr* were amplified from the immunoprecipitated chromatin DNA sample. Input = sonicated DNA fragments ranging from 100 to 500 bp in size; Sample = Δvfr/pBBR-vfr-3FLAG chromatin DNA immunoprecipitated with the anti-FLAG antibody; Mock = vfr/pBBR-3FLAG chromatin DNA immunoprecipitated with the anti-FLAG antibody (negative control).

Three putative target loci for Vfr in the proximity of the *phlF, phlG* and *vfr* were identified based on the ChIP-seq analysis. Primers for these three binding motifs were designed to verify the above results (Supplementary Table 2). 133 bp *phlF*, 134 bp *phlG*, and 162 bp *vfr* fragments could be PCR amplified from the ChIP sample (the chromatin DNA from strain Δvfr/pBBR-vfr-3FLAG obtained using the ANTI-FLAG antibody), similar to the amount obtained using the positive input control. Furthermore, Vfr binding motif is similar to that described in a previous study (Kanack et al., 2006). In contrast, the mock negative control (the chromatin DNA from strain Δvfr/pBBR-3FLAG obtained using the ANTI-FLAG antibody) failed to yield the corresponding PCR products when amplified under the same conditions (Figure 2). These results indicate that Vfr binds to the upstream regions of these three genes *in vivo*.

### Expression of Putative Vfr-Regulated Genes

**FIGURE 3:**
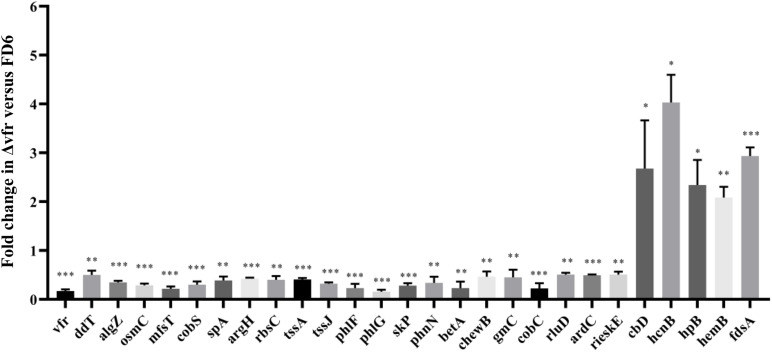
qPCR analysis of Vfr-dependent genes. RNA was isolated from wild-type and Δvfr cells, reverse-transcribed to cDNA and assayed by qPCR with gene-specific primers as described in the text. The experiments were performed in triplicate; average values ± standard deviations are shown ^∗^*P* < 0.05, ^∗∗^*P* < 0.01, and ^∗∗∗^*P* < 0.001.

The ChIP-seq analysis detected Vfr binding but did not demonstrate transcription of the downstream genes. To determine whether regulation of the identified target genes is Vfr-dependent, the expression of representative target genes was assessed by qPCR in both the wild-type and the Δvfr mutant. We observed that the expression of most target genes was significantly lower (*P* < 0.01) in the Δvfr mutant than in the wild-type strain, while the expression of 5 genes increased when the *vfr* gene was mutated (Figure 3). The downregulated genes included genes such as *tssJ* and *tssA*, which encode components of the type VI secretion system. The gene *phlF* encodes a transcriptional regulator that is involved in 2,4-DAPG biosynthesis, while *phlG* catalyzes the degradation of 2,4-DAPG to MAPG. The *phlG* gene is located immediately (46 bp) upstream of *phlF* and is divergently transcribed. Both of these genes exhibited significantly downregulated expression (*P* < 0.001) in the Δvfr mutant. Therefore, in subsequent experiments, we investigated the upstream regions of *phlF* and *phlG*, which are involved in 2,4-DAPG biosynthesis and regulation.

### Specific DNA-Vfr Interactions Determined With the Bacterial One-Hybrid Reporter System

**FIGURE 4:**
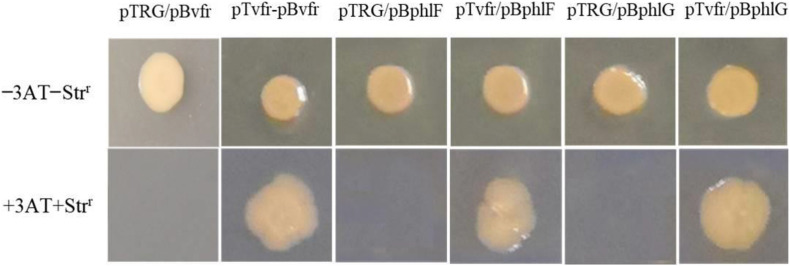
DNA-binding specificity determined using the bacterial one-hybrid reporter system. The upstream sequences of *vfr*, *phlF*, and *phlG* were individually cloned into pBXcmT. The *vfr* gene was cloned into the vector pTRG. A pair of pTvfr/pBvfr plasmids was cotransformed into the reporter strain *E. coli* XL1-Blue MRF’ Kan, and the growth of the strain was then tested on a selective medium containing 3-AT, Km^*r*^, Str^*r*^, and Cm^*r*^ as a positive control. The pTRG/pBvfr (pBphlF or pBphlG) plasmids were cotransformed into *E. coli* XL1-Blue MRF’ Kan as negative controls. Cotransformants with positive growth were selected on plates containing selective screening medium.

The bacterial one-hybrid reporter system is an efficient method for the detection of protein-DNA interactions *in vitro* and requires a positive growth cotransformant (Guo et al., 2009). To this end, we tested whether Vfr could bind to the upstream region of the *vfr* gene, as Vfr was reported to regulate its own expression in *P. aeruginosa* (Kanack et al., 2006; Fuchs et al., 2010). As shown in Figure 4, the purified Vfr protein bound to its own promoter, consistent with previous findings showing that the *vfr* gene is autoregulated. The sequence-specific interaction between Vfr and its promoter was used as a positive control in experiments designed to identify other Vfr target genes.

The upstream regions of *phlF* and *phlG* were amplified using specific primers (Supplementary Table 1) and inserted into the reporter vector pBXcmT. The *vfr* gene was cloned into pTRG. As shown in Figure 4, the strains with pTvfr/pBphlF or pTvfr/pBphlG grew as well as the positive cotransformant pTvfr/pBvfr, while those with pTRG/pBphlF or pTRG/pBphlG failed to grow. These results indicate that Vfr specifically interacts with the upstream regions of *phlF* and *phlG*.

### Vfr Interacts With Various Targets *in vitro*

**FIGURE 5:**
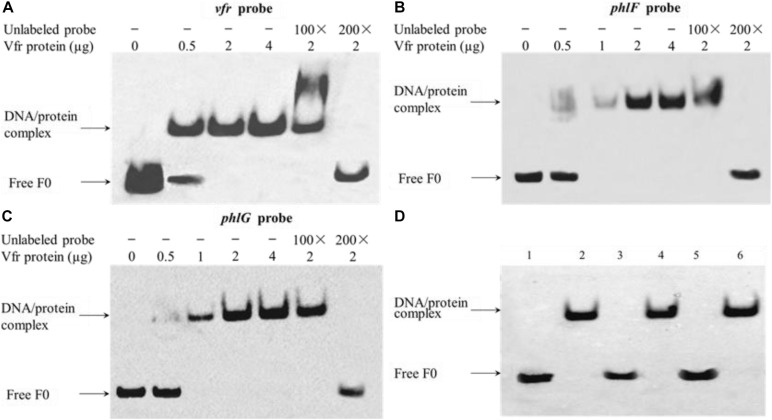
Vfr binds specifically to the *vfr*, *phlF* and *phlG* upstream regions. Recombinant Vfr protein was incubated with biotin-labeled probes **(A–C)** or with smaller probes **(D)** for 20 min before electrophoresis. The amount of Vfr used in the assay is shown above each gel. The amount of probe F0 used in each EMSA reaction was 0.02 μM; where indicated, a 100- to 200-fold excess of unlabeled probe was added to the reaction mixture prior to incubation. Lanes: (1) *vfr* probe alone; (2) *vfr* probe plus Vfr; (3) *phlF* probe alone; (4) *phlF* probe plus Vfr; (5) *phlG* probe alone; (6) *phlG* probe plus Vfr. Arrows indicate the positions of unbound free probes and Vfr-probe complexes.

To authenticate the putative Vfr binding regions identified in the *vfr*, *phlF* and *phlG* upstream regions, EMSAs were performed using 180- to 240-bp fragments of these sequences and purified recombinant Vfr (Supplementary Figure 3). The mobilities of the upstream fragments of *vfr*, *phlG* and *phlF* were partially or completely retarded in the presence of 0.5 and 2 μg of Vfr, respectively, suggesting that the regions corresponding to these fragments contain Vfr binding motifs. Moreover, competitive inhibition of Vfr binding to the upstream regions of *vfr*, *phlG* and *phlF* was observed when unlabeled probes were added to the reaction mixtures as competitors (Figures 5A–C). To further localize the Vfr binding sites, we synthesized three smaller (25-bp) fragments of the *vfr, phlF*, and *phlG* upstream regions and observed gel shift bands in the presence of these fragments. These results suggest that the 25-bp probes harbor consensus Vfr binding sites that are essential for Vfr binding to the upstream regions of *vfr*, *phlF*, and *phlG* (Figure 5D).

**FIGURE 6:**
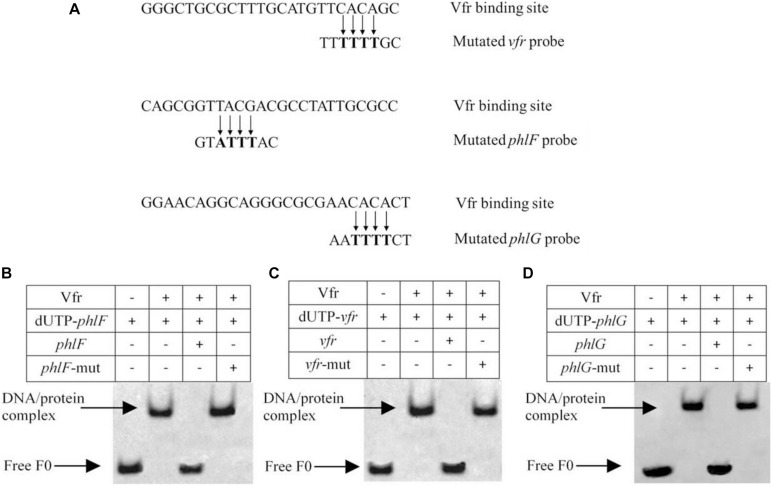
EMSA assay confirming the binding of Vfr to derivative probes of *vfr*, *phlF* and *phlG*. **(A)** Comparison of the Vfr binding consensus sequence of the target genes with the mutated Vfr binding sites used in EMSA. **(B–D)** For competition assays, a 200-fold excess of unlabeled wild-type or mutated oligonucleotide was added to the reaction mixture prior to the addition of 0.02 μM labeled probes.

The ChIP-seq analysis results indicated that the TCACA motif was highly conserved, and this potential Vfr consensus sequence was detected in the upstream regions of *vfr* (TCACA), *phlF* (TTACG) and *phlG* (ACACA). To verify the results of our analysis, EMSAs were performed using probes in which the sequences TCACA, TTACG, and ACACA were mutated to TTTTT, TATTT, and ATTTT, respectively (Figure 6A). As shown in Figures 6B–D, Vfr binding to the *vfr*, *phlF* and *phlG* probes was competed by unlabeled wild-type probes but not by the mutant probes. In this way, we localized Vfr binding within the upstream regions of these three genes to a 25-bp region that harbors only 5 bp of the consensus Vfr binding sequence. Taken together, these results indicate that Vfr specifically interacts with two novel binding sites identified by ChIP-seq, and does not require the previously known Vfr consensus motif.

## Discussion

CRP homologs (i.e., Clp and Vfr) act as important transcriptional regulators in many biocontrol agents and play important roles in the biosynthesis of antimicrobial compounds. For example, the *clp* gene in *L. enzymogenes* C3 was shown to globally regulate the expression of genes responsible for biocontrol traits, such as those that encode extracellular lytic enzymes or proteins involved in gliding motility and antimicrobial activity. Similar results were observed in *L. enzymogenes* OH11 (Kobayashi et al., 2005; Wang et al., 2014). In *P. chlororaphis* G05, Vfr is required for pyrrolnitrin biosynthesis but is not involved in the production of phenazine-1-carboxylic acid (Wu et al., 2019). These studies suggest that Crp homologs have evolved in such a way as to create species-specific mechanisms that affect the biosynthesis of antifungal compounds in various biocontrol agents. Although several studies have yielded insights into the function of Clp and Vfr, little is known regarding the specific role of Vfr in the regulation of antimicrobial metabolite production.

In the present study, we performed an *in vivo* ChIP-seq assay that identified 847 Vfr binding sites in the *P. protegens* genome. Our results show that Vfr directly or indirectly modulates the expression of a large number of genes in this bacterium. The majority of the genes whose expression is controlled by Vfr-bound regions are involved in metabolism, suggesting that Vfr plays important roles in the regulation of numerous metabolic pathways. Crp has been shown to have an effect on carbon metabolism in *E. coli* (Gosset et al., 2004). In this study, the transcript levels of most candidate genes were markedly decreased in the *vfr* deletion strain compared to the levels observed in the wild-type strain FD6 (Figure 3), suggesting that Vfr, similar to Crp, functions primarily as a positive regulator (Wang et al., 2014). However, some previously identified direct targets of Vfr, such as *vfr* and *rpoS*, were not identified in the present study. There are three possible reasons for this. First, the interaction between Vfr and these genes may be weak under the assay conditions we used (i.e., using the pBBR1MCS-2 vector and a mid-log phase culture). Myers et al. (2013) showed that FNR, a global regulator of anaerobiosis, control of certain target genes depends on a specific growth condition. Second, Vfr may require additional cofactors to bind these target genes. Transcription factors (TFs) rarely function alone, and it maybe common for combinatorial control by two or more TFs (Wade, 2015). Kleinman et al. (2017) supposed around 30% VjbR (a LuxR homolog) binding motifs did not show ChIP-seq signals due to additional elements working in the process of interaction of VjbR with its target genes in *Brucella abortus* 2308. Third, the presence of a C-terminal FLAG epitope tag may interfere with the ability to bind DNA (Myers, 2016). In the enteric pathogen *Salmonella enterica*, a lack of binding to some previously described OmpR-binding targets was shown to be due to the fact that the C-terminal FLAG tag affects binding to some OmpR-regulated genes (Perkins et al., 2013). These results suggest that ChIP-seq assays cannot be used to identify all Vfr binding sites.

The results of our previous study showed that Vfr regulates the biosynthesis of 2,4-DAPG and PLT, two previously characterized antifungal compounds produced by *P. protegens* (Zhang et al., 2016). To elucidate the precise molecular mechanism by which Vfr functions in *P. protegens* FD6, its complete genome was searched for the *P. aeruginosa* Vfr binding motif (TGNGANNNNNNTCACA) with nine conserved residues and 13 mismatches allowed. However, we did not identify the putative Vfr binding motif upstream of antibiotic-associated genes. These results suggest that Vfr may regulate these loci in an indirect way or that the Vfr binding motif is not conserved in all *Pseudomonas* species. Based on our ChIP-seq data, Vfr binding consensus motifs contain a TCACA sequence. In agreement with this result, previous studies have shown that the TCACA motif is the most important motif in the Vfr/cAMP-DNA interaction and that it is highly conserved in Vfr-dependent promoters (Kanack et al., 2006).

To validate the regulatory functions of Vfr in *P. protegens* FD6, we used RT-qPCR to measure the transcription levels of Vfr-associated genes. The results showed that the expression levels of most Vfr target genes were similar in the wild-type and the Δvfr mutant. There are two explanations for this result. First, Vfr activity may be associated with specific culture conditions. The LuxR-type regulator VjbR directly regulates the expression of certain *Brucella* virulence genes during the initial infection stages. The expression of other VjbR target genes may require specific environmental signals (Kleinman et al., 2017). Second, transcription factors typically function in coordination with other cofactors. The results of a previous genome-scale analysis of *E. coli* indicated that FNR may not bind directly to as much as half of the FNR-regulated operons due to the lack of a specific cofactor (Myers et al., 2013).

Previous studies of bacterial TFs have focused on TF binding sites located a short distance upstream of the genes they control. In *L. enzymogenes* OH11, the master regulator Clp controls twitching motility by binding directly to the *pilA* promoter region (Chen et al., 2018). The majority of TFs analyzed by ChIP-seq or ChIP-chip have been observed to bind intragenic sites that lie far from the annotated start codons of genes (Wade, 2015). For example, the binding sites of 50 TFs were investigated by ChIP-seq in *Mycobacterium tuberculosis*. Interestingly, most of the TF binding sites were located > 1 kb from the regulated genes (Galagan et al., 2013). Our ChIP-seq data also showed that the Vfr binding sites for both *phlF* and *phlG* are intragenic, although the results of subsequent analyses provided evidence for a direct interaction between Vfr and the upstream regions of these genes. Abbas et al. (2002) reported that *phlF* hinders the initiation of transcription of *phlACBD* through binding to the *phlA* promoter. Our RT-qPCR revealed that Vfr positively controls the expression of *phlF*. It may be that Vfr mutation releases the repressive effect of PhlF on expression of the *phlACBD* operon and promotes the synthesis of 2,4-DAPG. Vfr appears not to be the sole regulator of *phlG*, which encodes a hydrolase that degrades 2,4-DAPG to MAPG (Bottiglieri and Keel, 2006). Another TetR family regulator, *phlH*, which is located beween *phlF* and *phlG*, represses *phlG* expression by binding to its promoter region. Repression by *phlH* is relieved by 2,4-DAPG and MAPG (Yan et al., 2017). Further studies are required to understand how the interactions among Vfr, PhlF and PhlG affect the biosynthesis of 2,4-DAPG. To the best of our knowledge, this is the first report to show that Vfr binds to the upstream regions of *phlF* and *phlG*, and to describe a molecular mechanism that can explain the results of earlier studies of the role of Vfr in 2,4-DAPG biosynthesis. TF binding sites within genes are complex and may be associated with the regulation of other overlapping genes (Wade, 2015). Thus, subsequent studies will focus on whether or not other specific cofactors are involved in the regulatory networks for 2,4-DAPG biosynthesis.

In summary, the results of the present study provide a deeper understanding of the roles of Vfr in 2,4-DAPG synthesis, and the obtained ChIP-seq data will facilitate future investigation of the function of Vfr in the global regulation of *Pseudomonas* gene expression.

## Data Availability Statement

The datasets presented in this study can be found in online repositories. The names of the repository/repositories and accession number(s) can be found in the article/Supplementary Material.

## Author Contributions

XK, XC, and QZ conceived and designed the experiments. XK, CX, and CW conduced most of the experiments. QZ wrote the manuscript. All authors critically reviewed the manuscript.

## Conflict of Interest

The authors declare that the research was conducted in the absence of any commercial or financial relationships that could be construed as a potential conflict of interest.

## Supplementary Material

Supplementary Figure 1 Expression of the Vfr-FLAG fusion protein. The *vfr* deletion mutant strains containing either the empty vector or the Vfr-FLAG fusion construct were cultured in LB broth overnight, and cell extracts were then obtained. Equal amounts of cell extracts from the two mutants were resolved by SDS-PAGE and immunoblotted to assess Vfr-FLAG fusion construct expression levels. M, Prestained Color Protein Ladder; 1, Δvfr/pBBR; 2, Δvfr/pBBR-vfr-3FLAG.

Supplementary Figure 2 Analysis of Vfr-bound genomic regions. **(A)** Distribution of Vfr binding regions throughout the genome. **(B)** Distribution of Vfr binding regions within genes. **(C)** Inner circular plot of the *P. protegens* FD6 genome indicating ChIP-seq coverage and the positions of Vfr-regulated genes. The length of the red lines indicates the degree of enrichment.

Supplementary Figure 3 SDS-PAGE of purified pET-22b-His tagged Vfr protein from *E. coli* BL21. M, Protein standard marker; 1, pure Vfr protein.
