# Supplementary material for: ChIP-seq Analysis of the Global Regulator Vfr Reveals Novel Insights Into the Biocontrol Agent Pseudomonas protegens FD6
Source: Front Microbiol. 2021 May 14;12:667637. doi: 10.3389/fmicb.2021.667637 (PMC8160232; doi:10.3389/fmicb.2021.667637)
Supplement: Supplementary Figure 1 — Expression of the Vfr-FLAG fusion protein. The vfr deletion mutant strains containing either the empty vector or the Vfr-FLAG fusion construct were cultured in LB broth overnight, and cell extracts were then obtained. Equal amounts of cell extracts from the two mutants were resolved by SDS-PAGE and immunoblotted to assess Vfr-FLAG fusion construct expression levels. M, Prestained Color Protein Ladder; 1, Δvfr/pBBR; 2, Δvfr/pBBR-vfr-3FLAG. [file Image_1.pdf]

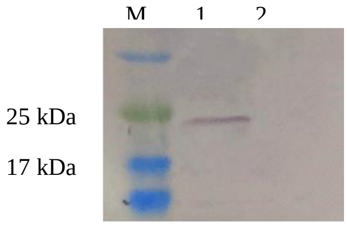

Supplementary Figure 1. Expression of the Vfr-FLAG fusion protein. The *vfr* deletion mutant strains containing either the empty vector or the Vfr-FLAG fusion construct were cultured in LB broth overnight, and cell extracts were then obtained. Equal amounts of cell extracts from the two mutants were resolved by SDS-PAGE and immunoblotted to assess Vfr-FLAG fusion construct expression levels. M, Prestained Color Protein Ladder; 1, Δvfr/pBBR; 2, Δvfr/pBBR-vfr-3FLAG
