# Supplementary material for: ChIP-seq Analysis of the Global Regulator Vfr Reveals Novel Insights Into the Biocontrol Agent Pseudomonas protegens FD6
Source: Front Microbiol. 2021 May 14;12:667637. doi: 10.3389/fmicb.2021.667637 (PMC8160232; doi:10.3389/fmicb.2021.667637)
Supplement: Supplementary Figure 2 — Analysis of Vfr-bound genomic regions. (A) Distribution of Vfr binding regions throughout the genome. (B) Distribution of Vfr binding regions within genes. (C) Inner circular plot of the P. protegens FD6 genome indicating ChIP-seq coverage and the positions of Vfr-regulated genes. The length of the red lines indicates the degree of enrichment. [file Image_2.pdf]

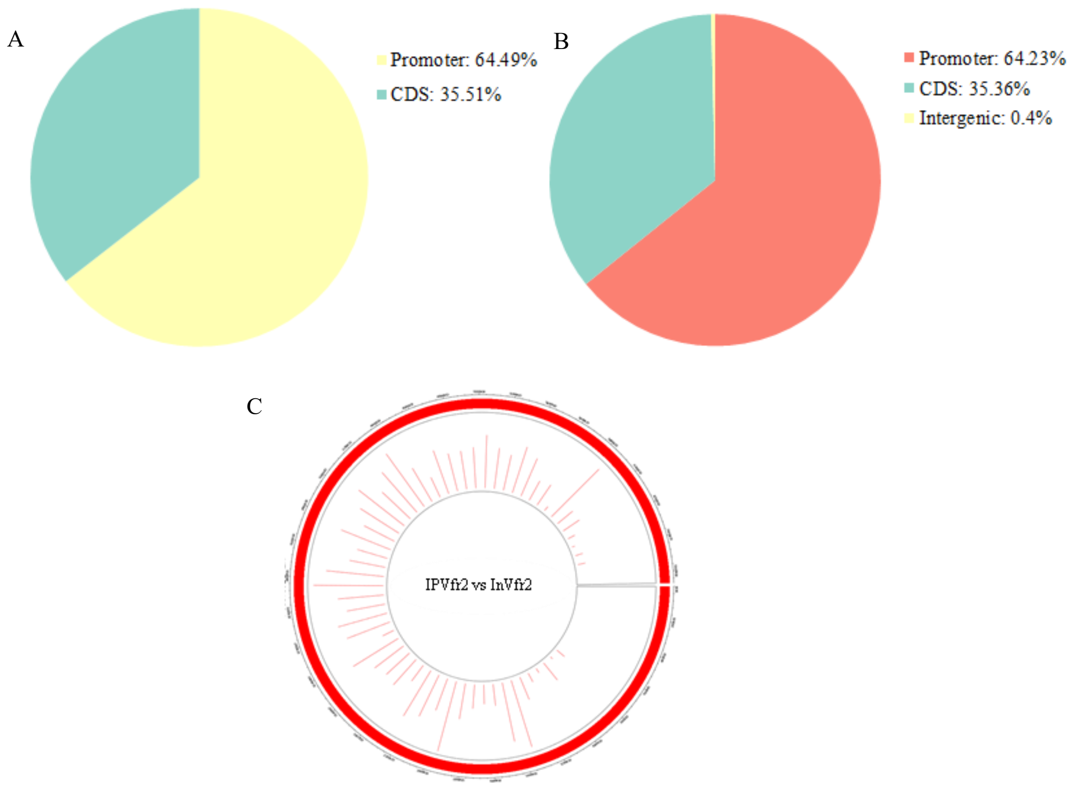

Supplementary Figure 2. Analysis of Vfr-bound genomic regions. (A) Distribution of Vfr binding regions throughout the genome. (B) Distribution of Vfr binding regions within genes. (C) Inner circular plot of the *P. protegens* FD6 genome indicating ChIP-seq coverage and the positions of Vfr-regulated genes. The length of the red lines indicates the degree of enrichment.
