# Supplementary material for: ChIP-seq Analysis of the Global Regulator Vfr Reveals Novel Insights Into the Biocontrol Agent Pseudomonas protegens FD6
Source: Front Microbiol. 2021 May 14;12:667637. doi: 10.3389/fmicb.2021.667637 (PMC8160232; doi:10.3389/fmicb.2021.667637)
Supplement: Supplementary Figure 3 — SDS-PAGE of purified pET-22b-His tagged Vfr protein from E. coli BL21. M, Protein standard marker; 1, pure Vfr protein. [file Image_3.pdf]

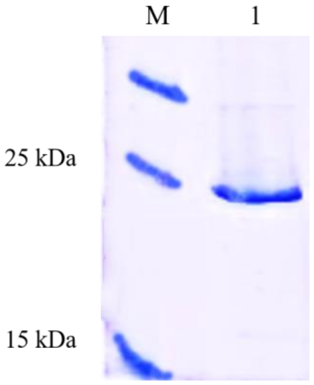

Supplementary Figure 3. SDS-PAGE of purified pET-22b-His tagged Vfr protein from *E. coli* BL21. M: Protein standard marker; 1: pure Vfr protein.
